# Extracellular chloride is required for efficient platelet aggregation

**DOI:** 10.1080/09537104.2017.1332367

**Published:** 2017-07-20

**Authors:** Kirk A. Taylor, Darren G. S. Wilson, Matthew T. Harper, Nicholas Pugh

**Affiliations:** ^a^ Department of Biomedical and Forensic Sciences, Anglia Ruskin University, Cambridge, UK; ^b^ Department of Pharmacology, Cambridge University, Cambridge, UK

**Keywords:** ADP, aggregation, chloride, ion channels platelets, thrombin

## Abstract

Anion channels perform a diverse range of functions and have been implicated in ATP release, volume regulation, and phosphatidylserine exposure. Platelets have been shown to express several anion channels but their function is incompletely understood. Due to a paucity of specific pharmacological blockers, we investigated the effect of extracellular chloride substitution on platelet activation using aggregometry and flow cytometry. In the absence of extracellular chloride, we observed a modest reduction of the maximum aggregation response to thrombin or collagen-related peptide. However, the rate of aggregation was substantially reduced in a manner that was dependent on the extracellular chloride concentration and aggregation in the absence of chloride was noticeably biphasic, indicative of impaired secondary signaling. This was further investigated by targeting secondary agonists with aspirin and apyrase or by blockade of the ADP receptor P2Y12. Under these conditions, the rates of aggregation were comparable to those recorded in the absence of extracellular chloride. Finally, we assessed platelet granule release by flow cytometry and report a chloride-dependent element of alpha, but not dense, granule secretion. Taken together these data support a role for anion channels in the efficient induction of platelet activation, likely via enhancement of secondary signaling pathways.

## Introduction

In contrast to the role of cations (i.e., Ca^2+^, K^+^, and Zn^2+^) [1–3], the contribution(s) of anions to platelet activation remains unclear. Anion channels perform diverse functions including regulatory volume decrease [4], phosphatidylserine exposure [5], and ATP release [6]. Early patch clamp recordings demonstrated functional expression of Ca^2+^-activated Cl^−^ channels in platelets [7, 8] and a megakaryocyte-like DAMI cell line [9]. Proteomic [10] and transcriptomic [11] analyses have since identified numerous anion channels that may be expressed by platelets. Of these, functional expressions of CLIC1 (Intracellular Cl^−^ channel-1) [12], TMEM16F [13], and pannexin-1 [14] have been confirmed. Indicative of a hemostatic role for anion channels, CLIC1- and TMEM16F-deficient mice have associated platelet-related bleeding phenotypes [12, 13]. Additionally, pannexin-1 channels have been shown to amplify platelet activation responses to threshold agonist concentrations [14]. These ATP-permeable channels are associated with inflammatory conditions and may contribute to atherosclerosis [15].

Given the lack of specific anion channel blockers, we focus on the effect of extracellular Cl^−^ ([Cl^−^]_o_) substitution on platelet activation. Our experiments highlight a role for anion channels in modulating the rate of platelet aggregation.

## Methods

### Materials

Aspirin, apyrase, and thrombin were from Sigma (Poole, UK). AR-C66096 was from Tocris Bioscience (Bristol, UK). Cross-linked collagen-related peptide (CRP-XL) was prepared as described previously [16] and supplied by R. Farndale (Cambridge, UK). Unless indicated, all other reagents were from Sigma.

### Washed platelet preparation

This study was approved by the local Ethics Committee at Anglia Ruskin University. Human blood was collected from healthy volunteers following informed consent in accordance with the Declaration of Helsinki. Blood was collected into 11 mM sodium citrate and washed platelets were prepared as described previously [17]. Platelets were resuspended in a nominally calcium-free buffer containing (in mM) 145 NaCl, 5 KCl, 1 MgCl_2_, 10 glucose, 10 HEPES, titrated to pH 7.35 with NaOH. Where indicated, [Cl^−^]_o_ was substituted by equimolar gluconate.

### Aggregometry

**Figure 1.**
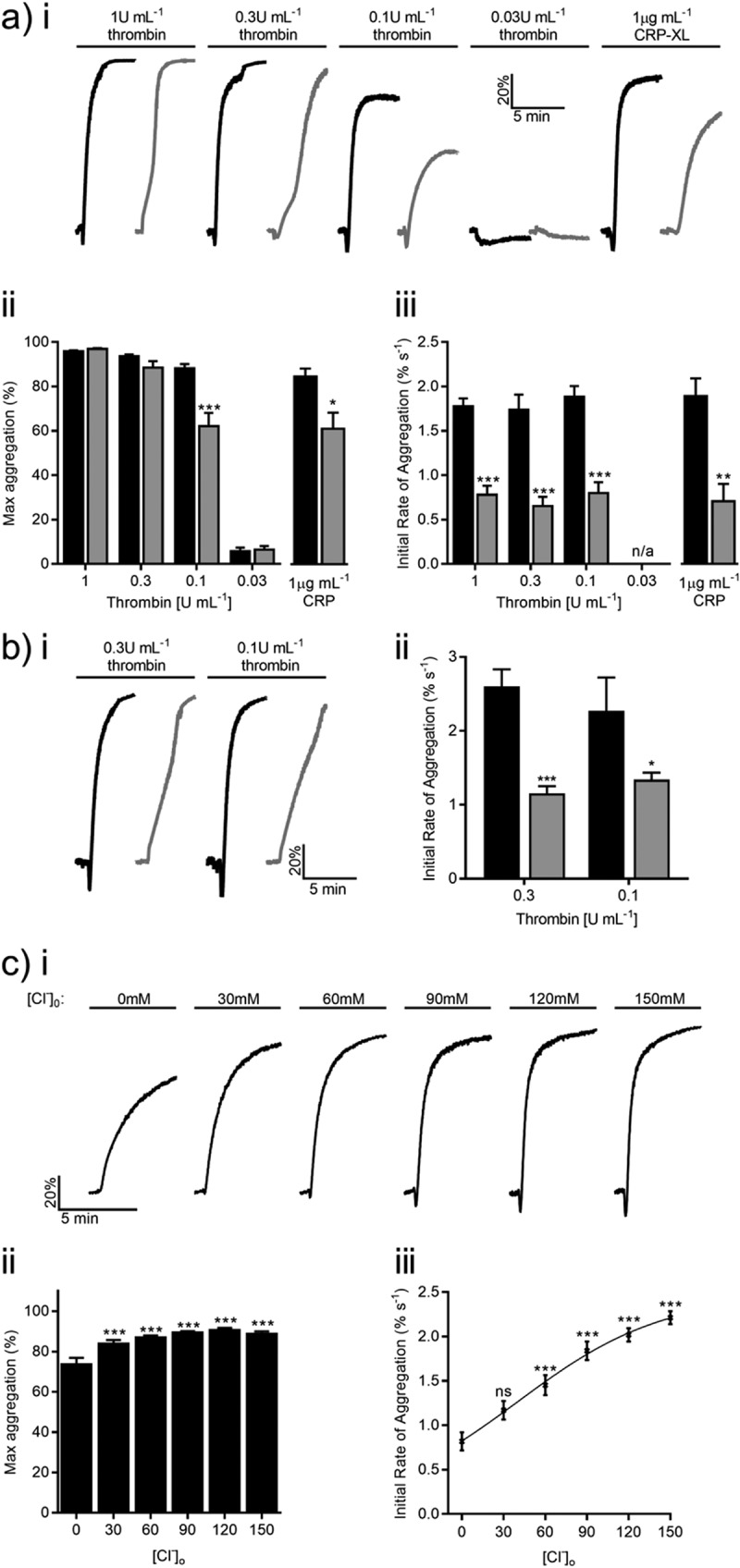
Extracellular chloride is required for efficient platelet activation. (a) Washed platelets were stimulated by increasing concentrations of thrombin or 1 µg mL^−1^ CRP-XL in the presence of 151 mM (black) or 1 mM (gray) [Cl^−^]_o_. Representative aggregation traces are shown for each condition (ai). Maximum aggregation (aii) and the initial rate of aggregation (aiii) were calculated for each condition. (b) In the presence of 2 mM [Ca^2+^]_o_, [Cl^−^]_o_ substitution continued to reduce the initial rate of thrombin-evoked aggregation. (c) The sensitivity of thrombin-evoked (0.1 U mL^−1^) aggregation to Cl^−^ was assessed by increasing [Cl^−^]_o_ from 1 to 151 mM in 30 mM increments. Representative traces (ci), maximum (cii) and initial rate of thrombin-induced (0.1 U mL^−1^) aggregation (ciii) for each [Cl^−^]_o_ are shown. The rate of aggregation increased across the concentration range with an EC_50_ = 46.5 ± 23.3 mM. Data are representative of a minimum of four independent experiments. Thrombin and CRP-XL data sets were analyzed by two-way ANOVA and Student’s *t* test, respectively.

Platelet aggregation was monitored as described previously using an AggRam aggregometer (Helena Biosciences, Gateshead, UK) [17]. In the experiments of Figure 1c, aliquots of 151 and 1 mM [Cl^−^]_o_-containing platelet suspensions were mixed 5 min prior to agonist addition. Platelets were preincubated with each drug(s) for 5 min at 37°C.

### Granule release

Thrombin-evoked alpha and dense granule release was assessed by flow cytometry using fluorescently conjugated CD62P and CD63 antibodies (BD Biosciences, Oxford, UK), respectively. Antibody binding was monitored for 5 min using an Accuri C6 Flow cytometer (BD Biosciences) and the percentage of positive cells was calculated within FlowJo (V10.2, Oregon, USA).

### Data analysis and statistics

Maximum aggregation (%) and the initial rate of aggregation (% s^−1^) were calculated in Excel (Microsoft, Redmond, Washington, USA), where rate was determined as the change in aggregation (%) in the first 30 s following shape change. Data were analyzed in GraphPad Prism by two-way ANOVA or Student’s *t* test as indicated and are representative of a minimum of four independent experiments. ***, **, *, and ns denote *P *< 0.001, *P* < 0.01, *P* < 0.05, and not significant, respectively.

## Results

Platelet aggregation was assessed in the presence/absence of [Cl^−^]_o_ in response to increasing thrombin concentrations (Figure 1a). Removal of [Cl^−^]_o_ did not affect the maximum aggregation recorded across a 5-min time course in response to 0.03, 0.3, or 1 U mL^−1^ thrombin (Figure 1aii). A reduction from 82.3 ± 1.9% to 62.2 ± 6.0% (*P* < 0.001, Figure 1aii) was observed at an intermediate concentration (0.1 U mL^−1^). Cl^−^ substitution affected the kinetics of platelet activation as the aggregation rate decreased by 1.1 ± 0.2%, 1.1 ± 0.2%, and 1.0 ± 0.2% s^−1^ in response to stimulation by 0.1, 0.3, and 1 U mL^−1^ thrombin, respectively (*P* < 0.01, Figure 1aiii). Similar effects were observed in the presence of 2 mM Ca^2+^ (Figure 1b), suggesting that this effect was not due to Ca^2+^ buffering. This observation was not exclusive to thrombin-evoked aggregation; removal of [Cl^−^]_o_ reduced maximal CRP-XL-induced (1 µg mL^−1^) aggregation by 23.5 ± 3.9% (*P* < 0.01, Figure 1aii) and the rate decreased by 1.2 ± 0.1% s^−1^ (*P* < 0.001, Figure 1aiii). The sensitivity of thrombin-evoked (0.1 U mL^−1^) aggregation to Cl^−^ was assessed by increasing [Cl^−^]_o_ from 1 to 151 mM (30 mM increments; Figure 1c). There was no change in maximum aggregation beyond 31 mM [Cl^−^]_o_ yet rates increased across the concentration range (EC_50_ = 46.5 ± 23.3 mM).

**Figure 2.**
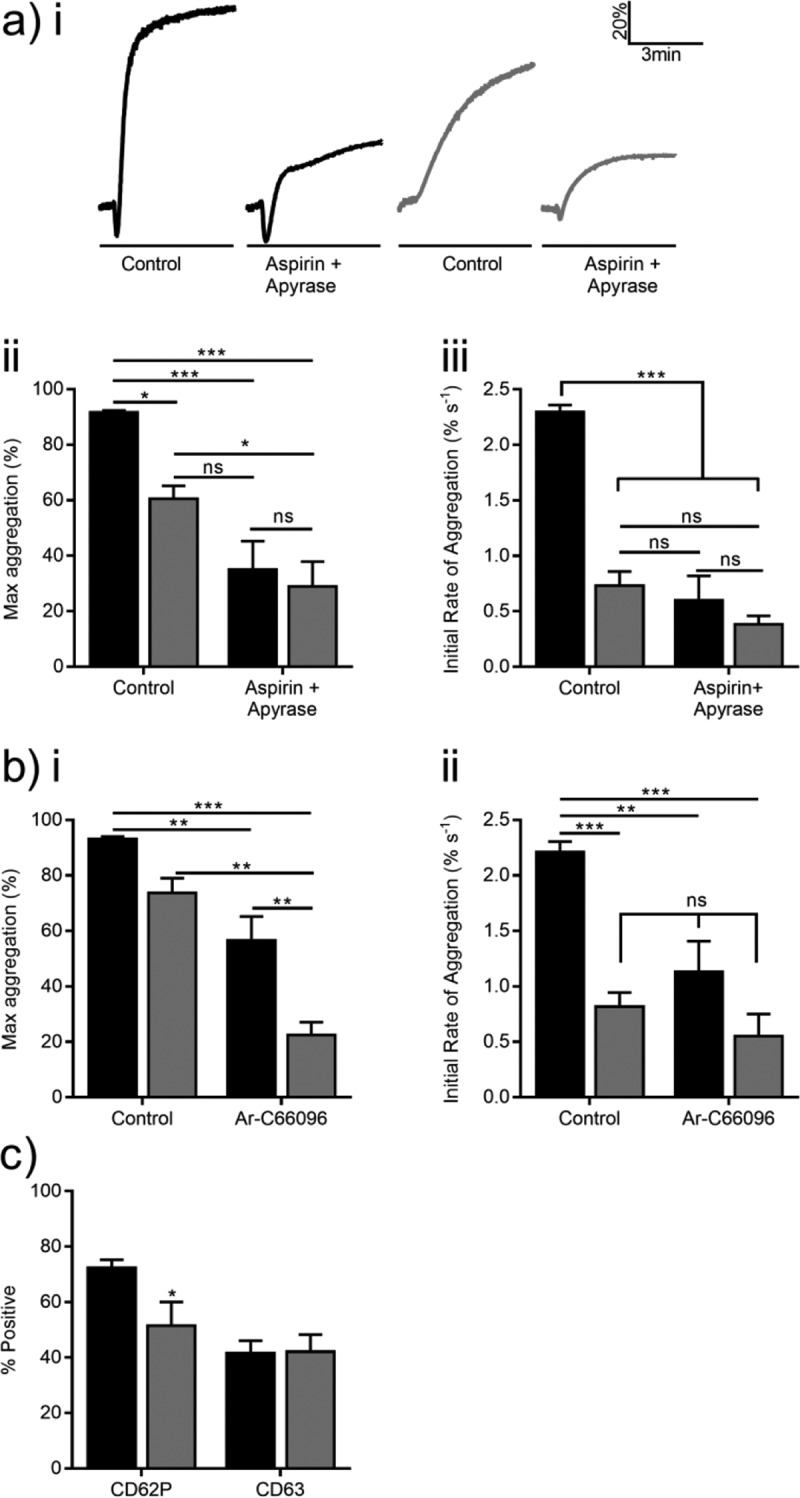
Role for secondary signaling during Cl^–^-dependent platelet aggregation. (a) Washed platelets were preincubated with 100 µM aspirin (cyclooxygenase inhibitor) and 5 U mL^−1^ apyrase (ectonucleotidase) prior to performing aggregometry in the presence of 151 mM (black) or 1 mM (gray) [Cl^−^]_o_. Representative traces (ai), maximum (aii), and initial rate (aiii) of thrombin-induced (0.1 U mL^−1^) aggregation are shown in the presence of vehicle control (0.1% ethanol) or aspirin plus apyrase. (b) Summary data for maximum (bi) and initial rate of thrombin-evoked (0.1 U mL^−1^) platelet aggregation (bii) in the presence of vehicle control (H_2_O) or 1 µM Ar-C66096 (P2Y12 inhibitor). (c) Alpha (i) and dense (ii) granule release before (unstimulated) and after 0.1 U mL^−1^ thrombin stimulation was assessed by flow cytometry using fluorescently labeled CD62P and CD63 antibodies, respectively. Data are representative of a minimum of four independent experiments and data were analyzed by two-way ANOVA.

Differences in aggregation rate may be due to reduced secondary signaling in the absence of [Cl^−^]_o_. Platelets were preincubated with 100 µM aspirin and 5U mL^−1^ apyrase to assess contributions by thromboxane A_2_ and extracellular nucleotides. These compounds reduced maximum aggregation in response to 0.1 U mL^−1^ thrombin by 56.6 ± 10.2% (*P* < 0.001) and 31.6 ± 10.2% (*P* < 0.05, Figure 2ai, ii) in the presence of 151 and 1 mM [Cl^−^]_o_, respectively. The rate of platelet aggregation following aspirin and apyrase treatment in the presence of 151 mM (0.6 ± 0.2% s^−1^) or 1 mM (0.38 ± 0.08% s^−1^) [Cl^−^]_o_ was comparable to that of 1 mM [Cl^−^]_o_ control (0.7 ± 0.1% s^−1^; *P* > 0.05, Figure 2aiii).

P2Y12-mediated signaling is a major step during integrin α_IIb_β_3_ activation [18, 19] and reduced ADP availability may account for the observed aggregation defect. 0.1 U mL^−1^ thrombin-evoked aggregation in the presence of a P2Y12 inhibitor (1 µM AR-C66096) decreased by 36.7 ± 8.2% and 51.2 ± 7.4% in the presence of 151 and 1 mM [Cl^−^]_o_ (*P* < 0.01, Figure 2bi), respectively. It is noteworthy that no differences between the aggregation rate of AR-C66096-treated platelets in 151 or 1 mM [Cl^−^]_o_ and the 1 mM [Cl^−^]_o_ control were observed (*P* > 0.05, Figure 2bii), suggesting a role for P2Y12 during Cl^–^-dependent aggregation. Finally, we assessed thrombin-induced granule release by flow cytometry. Peak alpha granule release was reduced from 72.4 ± 2.9% to 59.9 ± 1.6% (*P *< 0.001, Figure 2c), while dense granule release was unaffected by Cl^−^ substitution (*P* > 0.05, Figure 2c).

## Conclusions

Here we demonstrate that [Cl^−^]_o_ enhances the rate of platelet aggregation in a concentration-dependent manner (Figure 1). This effect was equivalent to blockade of secondary mediators and P2Y12 inhibition (Figure 2). One possible explanation of these data is that [Cl^−^]_o_ is required for efficient release of ATP and/or ADP from the platelet, but we failed to observe a change in dense granule secretion (Figure 2c). Pannexin-1 has been shown to activate in response to elevation of intracellular Ca^2+^ ([Ca^2+^]_i_) [20], facilitating cytosolic ATP release [14]. It has been suggested that ADP release may occur via a similar mechanism [21]. Given that release of alpha granule cargo (e.g., fibrinogen, thrombin, and Zn^2+^) is required for aggregation and is enhanced by P2Y12 signaling [22–24], it is possible that [Cl^−^]_o_ enhances platelet activation by mediating efficient alpha granule secretion.

Reduction of [Cl^−^]_o_ has been shown to substantially reduce thrombin-plus-CRP-XL-mediated elevation of [Ca^2+^]_i_ in a similar manner to that of Cl^−^ channel blockers [25]. It has been suggested that Cl^−^ currents hyperpolarize the cell, increasing driving force for Ca^2+^ influx [9]. However, the platelet Cl^−^ equilibrium is ≈35 mV in the platelet [7], meaning activation of a Cl^−^ conductance would depolarize rather than hyperpolarize platelets. Reduced Ca^2+^ influx may be due to reduced secondary signaling, rather than altered membrane potential.

We have focused on the contribution of [Cl^−^]_o_ by way of ionic substitution experiments because of the paucity of specific pharmacological tools to study anion channels. This may also explain why anion channels have previously received much less attention than cation channels. It is worth noting that anion channels have been associated with cystic fibrosis, bleeding phenotypes, and inflammatory conditions [12, 13, 15, 26, 27] and may represent valuable therapeutic targets, as demonstrated by clinical use of CFTR modulators [28]. Further work will be required to investigate the contribution(s) by the cohort of platelet anion channels during platelet activation.
